# Insights Into Mucosal-Associated Invariant T Cell Biology From Studies of Invariant Natural Killer T Cells

**DOI:** 10.3389/fimmu.2018.01478

**Published:** 2018-06-28

**Authors:** Lucy C. Garner, Paul Klenerman, Nicholas M. Provine

**Affiliations:** ^1^Translational Gastroenterology Unit, Nuffield Department of Medicine, University of Oxford, Oxford, United Kingdom; ^2^Peter Medawar Building for Pathogen Research, University of Oxford, Oxford, United Kingdom

**Keywords:** mucosal-associated invariant T cells, natural killer T cells, innate-like T cells, phenotype, development, activation, effector function, subsets

## Abstract

Mucosal-associated invariant T (MAIT) cells and invariant natural killer T (iNKT) cells are innate-like T cells that function at the interface between innate and adaptive immunity. They express semi-invariant T cell receptors (TCRs) and recognize unconventional non-peptide ligands bound to the MHC Class I-like molecules MR1 and CD1d, respectively. MAIT cells and iNKT cells exhibit an effector-memory phenotype and are enriched within the liver and at mucosal sites. In humans, MAIT cell frequencies dwarf those of iNKT cells, while in laboratory mouse strains the opposite is true. Upon activation *via* TCR- or cytokine-dependent pathways, MAIT cells and iNKT cells rapidly produce cytokines and show direct cytotoxic activity. Consequently, they are essential for effective immunity, and alterations in their frequency and function are associated with numerous infectious, inflammatory, and malignant diseases. Due to their abundance in mice and the earlier development of reagents, iNKT cells have been more extensively studied than MAIT cells. This has led to the routine use of iNKT cells as a reference population for the study of MAIT cells, and such an approach has proven very fruitful. However, MAIT cells and iNKT cells show important phenotypic, functional, and developmental differences that are often overlooked. With the recent availability of new tools, most importantly MR1 tetramers, it is now possible to directly study MAIT cells to understand their biology. Therefore, it is timely to compare the phenotype, development, and function of MAIT cells and iNKT cells. In this review, we highlight key areas where MAIT cells show similarity or difference to iNKT cells. In addition, we discuss important avenues for future research within the MAIT cell field, especially where comparison to iNKT cells has proven less informative.

## Introduction

Mucosal-associated invariant T (MAIT) cells and invariant natural killer T (iNKT) cells are two populations of innate-like T cells that have emerged in recent years as crucial players in the development and maintenance of immunity. This is demonstrated by the array of infectious, inflammatory, and malignant diseases in which they have been implicated and in which they play diverse roles (1–5). Depending on the nature of the infectious or inflammatory setting, these can range from host protective functions, for example, antimicrobial or antitumor responses, to the augmentation of disease (1–5).

Mucosal-associated invariant T cells and iNKT cells function at the bridge between innate and adaptive immunity. While they express a T cell receptor (TCR), similar to conventional T cells of the adaptive immune system, their TCRs are semi-invariant and recognize a limited range of non-peptide ligands presented by monomorphic MHC-like molecules (6, 7). Consequently, the TCRs of MAIT cells and iNKT cells may function in a manner more akin to that of the pattern-recognition receptors expressed on innate immune cells, for example, macrophages and dendritic cells (DCs). Furthermore, MAIT cells and iNKT cells display an effector-memory phenotype prior to antigen exposure, can be activated by cytokines independent of their TCR, and can rapidly exert their effector functions upon activation without the requirement for clonal expansion, properties more analogous to innate immune cell types (8, 9). Given these distinctive characteristics, they are likely to play particularly important roles during the early stages of an immune response, prior to the differentiation of conventional effector T cells.

Although MAIT cells and iNKT cells exhibit many similarities, they also show important differences that are often disregarded. For instance, MAIT cells are the largest subset of donor-unrestricted T cells in humans, and their frequency in peripheral blood and certain tissues can be more than 100-fold greater than that of iNKT cells, whereas in mice iNKT cells are the more abundant population in most tissues (10, 11). Moreover, while MAIT cells in humans form a homogeneous population with mixed Th1/Th17 functionality, iNKT cells are highly diverse and can be divided into functionally distinct subsets (5, 11).

Given their much higher frequency in mice and the earlier availability of tetramers for their specific identification, iNKT cells have been more widely studied than MAIT cells. Furthermore, because of the similarities in their phenotypes, findings from the iNKT cell field have often been assumed to also apply to MAIT cells. With the discovery of MAIT cell ligands and the recent generation of tetramers for accurate MAIT cell identification (7, 12, 13), it is timely to consider the phenotype, development, and function of MAIT cells, how this relates to iNKT cells, and where gaps remain in our understanding. This review will focus on key areas of similarity and difference between MAIT cells and iNKT cells and will highlight important remaining questions in the MAIT cell field, many of which should now be feasible to address using the newly available tetramers.

## Key Characteristics

### Frequency and Localization

Mucosal-associated invariant T cells represent a relatively large population of lymphocytes in humans, comprising up to 10% of peripheral blood T cells (14). iNKT cells are comparatively rare, with an average frequency of around 0.1% of T cells, although both MAIT and iNKT cell frequencies are highly variable between individuals (15–17). Interestingly, iNKT cells are far more abundant than MAIT cells in mice (18, 19).

Mucosal-associated invariant T cells preferentially localize to peripheral tissues (11), analogous to iNKT cells (10). In humans, MAIT cells are particularly enriched in the liver (5–50% of T cells) and are also abundant in adipose tissue, in the lung, in the female genital tract, and to varying degrees in the gut, while their frequency is low in peripheral lymph nodes (12, 14, 20–26). MAIT cells and iNKT cells in mice show a largely similar tissue distribution to human MAIT cells, with enrichment in the liver and lung (18, 19). Due to their low abundance, the tissue distribution of human iNKT cells remains poorly characterized, although they are particularly enriched in adipose tissue (27), comparable to human MAIT cells (20).

Evidence from parabiosis studies in mice suggests that iNKT cells comprise predominantly tissue-resident populations that do not recirculate, in contrast to conventional CD4^+^ and CD8^+^ T cells (28, 29). The capacity of tissue MAIT cells to recirculate has not yet been examined. In support of a tissue-resident phenotype, MAIT cells lack expression of the lymph node homing receptors CD62L and CCR7 (14) and express tissue-resident T cell markers in mucosal tissue, including CD69, CD103, and CRTAM (25, 30). In addition, human liver MAIT cells express LFA-1 (31), a molecule that is required for retention of liver iNKT cells in mice (28). MAIT cells accumulate in the lungs of mice following intranasal infection with *Salmonella enterica* serovar Typhimurium and remain *in situ* for at least 7 weeks post-infection, implying long-term retention in tissues (32). Finally, MAIT cells express the transcription factor PLZF (33), and conventional CD4^+^ T cells in mice acquire a tissue-resident phenotype following ectopic expression of PLZF (28). However, CCR7^−^CD103^−^ MAIT cells have recently been identified in human thoracic duct lymph at a similar frequency to that in peripheral blood (34). As CCR7 is required for lymph node entry, the authors suggest that MAIT cells in the lymph must have exited from non-lymphoid tissues. Based on these findings, it is possible that tissue MAIT cells comprise largely resident populations, while MAIT cells in certain tissues and/or particular subsets, are capable of recirculation. Such a model would need to be tested in mouse parabiosis experiments.

In mice, MAIT cell frequency is under considerable genetic control. MAIT cells show differential abundance in different strains of mice (19), and increased MAIT cell numbers in CAST/EiJ mice can be mapped to a single genetic locus (35). Similarly, iNKT cell frequency is strongly regulated by genetic factors, as indicated by longitudinal and twin studies in humans, and analyses of iNKT cell frequency in different wild-type and congenic mouse strains (36–40). In addition to genetics, MAIT cell frequency is influenced by a number of environmental factors. Their frequency decreases in the blood with age (after ~25 years old) and in numerous diseases, while they expand in certain tissues upon infection or inflammation (3, 32, 41–44), comparable to iNKT cells (10, 45, 46). Moreover, the frequency of Vα7.2^+^CD161^hi^ T cells (a proxy for MAIT cell frequency) shows no correlation in human mothers and neonates, and the correlation in Vα7.2^+^CD161^hi^ T cell frequency at birth is equally high in monozygotic and dizygotic twins (47). This suggests that environmental factors may dominate over genetic factors in regulating MAIT cell frequency in humans. However, these findings need to be confirmed using the MR1/5-OP-RU [5-(2-oxopropylideneamino)-6-d-ribitylaminouracil] tetramer for MAIT cell identification, as MR1/5-OP-RU tetramer^+^ MAIT cells comprise only a small fraction (<20%) of Vα7.2^+^CD161^hi^ T cells at birth, in contrast to adults, where Vα7.2^+^CD161^hi^ T cells are typically >95% MR1/5-OP-RU tetramer^+^ (47). Therefore, further research is required to establish the relative role of genetic and environmental factors in regulating MAIT cell frequency in mice and humans.

### TCR Usage

The semi-invariant αβ TCRs of MAIT cells and iNKT cells comprise a largely invariant TCRα chain paired with a biased repertoire of Vβ chains. In humans, MAIT cells express a Vα7.2-Jα33/12/20 (TRAV1-2/TRAJ33/12/20) TCRα chain preferentially paired with Vβ2 or Vβ13 (TRBV20 or TRBV6) (12, 48–50), while the iNKT TCR comprises a Vα24-Jα18 (TRAV10/TRAJ18) TCRα chain paired exclusively with Vβ11 (TRBV25) (Table 1) (48, 51, 52). Conventional T cells recognize short peptide antigens presented by highly polymorphic MHC Class I or MHC Class II molecules. By contrast, MAIT cells and iNKT cells recognize non-peptide ligands bound to monomorphic MHC Class I-like molecules, namely riboflavin metabolites bound to MR1 (7, 13, 22), and glycolipid/phospholipid antigens bound to CD1d (6), respectively (Table 1).

**Table 1:** Characteristics of human and mouse mucosal-associated invariant T (MAIT) and invariant natural killer T (iNKT) cells.

	T cell receptor (TCR) usage	MHC-like molecule	Ligands	CD4/CD8	Naïve/memory	Chemokine receptors	Cytokine receptors	Cytokines produced	Transcription factors
**Human MAIT**	Vα7.2-Jα33/12/20 (TRAV1-2/TRAJ33/12/20) TCRα. Biased repertoire of Vβ chains – Vβ2, Vβ13 (TRBV20, TRBV6). Oligoclonal CDR3β	MR1	*Activating* – riboflavin metabolites (5-OP-RU, 5-OE-RU), drug metabolites (e.g., diclofenac metabolites). *Inhibitory* – folic acid metabolites (6-FP, Ac-6-FP), small organic molecules (e.g., salicyclates)	70–85% CD8^+^ (~50% CD8α^+^α^+^, ~50% CD8α^+^β^+^), 10–20% DN, <5% CD4^+^	CD45RO^+^ CCR7^−^CD62L^lo^ effector-memory	CCR2, CCR5, CCR6, CXCR6	IL-12R, IL-18R, IL-7R, IL-15R, IL-23R	IFN-γ, TNF-α, IL-17A, MIP-1β	PLZF^+^, RORγt^+^, T-bet^int^, Eomes^hi^, Helios^int^

**Mouse MAIT**	Vα19-Jα33 (TRAV1/TRAJ33) TCRα. Biased repertoire of Vβ chains – Vβ6, Vβ8 (TRBV19, TRBV13)	MR1	*Activating* – riboflavin metabolites (5-OP-RU, 5-OE-RU). *Inhibitory* – folic acid metabolites (6-FP, Ac-6-FP), small organic molecules (e.g., salicyclates)	B6 mice – 55–90% DN, <25% CD8^+^ (~50% CD8α^+^β^−^, ~50% CD8α^+^β^+^), <20% CD4^+^. Variation across tissues	CD44^hi^CD62L^lo^ effector-memory	CXCR6	IL-18R, IL-7R	IFN-γ, IL-17A, MIP-1α	PLZF^+^, RORγt^+^ (subset), T-bet^+^ (subset)

**Human iNKT**	Vα24-Jα18 (TRAV10/TRAJ18), Vβ11 (TRBV25). Diverse CDR3β	CD1d	*Activating* – glycolipids (e.g., glycosphingolipids, diacylglycerols, cholesteryl α-glucosides) and phospholipids (including lysophospholipids, e.g., ether-bonded mono-alkyl glycerophosphates)	10–100% CD4^−^ (average = 60%; ~50% CD8^+^ [>95% CD8α^+^α^+^], ~50% DN), 0–90% CD4^+^ (average = 40%)	Predominantly CD45RO^+^ CCR7^−^CD62L^lo^ effector-memory	Majority express CXCR3, CXCR4, CCR2, CCR5. Subset-specific: *CD4^+^* – CCR4. *CD4*^−^ – CXCR6, CCR6, CCR1	IL-12R, IL-18R, IL-7R, IL-15R	MIP-1α, MIP-1β. *CD4^+^* – IFN-γ, TNF-α, IL-4, IL-13, IL-10, GM-CSF. *CD4*^−^ – IFN-γ, TNF-α	PLZF^+^, T-bet^+^ (~50%), Eomes^+^ (~30%), FoxP3^+^ (*in vitro*)

**Mouse iNKT**	Vα14-Jα18 (TRAV11/TRAJ18). Biased repertoire of Vβ chains – Vβ8.2, Vβ7, Vβ2 (TRBV13-2, TRBV29, TRBV1). Diverse CDR3β	CD1d	*Activating* – glycolipids (e.g., glycosphingolipids, diacylglycerols, cholesteryl α-glucosides) and phospholipids (including lysophospholipids, e.g., ether-bonded mono-alkyl glycerophosphates)	B6 mice – 60–80% CD4^+^, 20–40% DN. Variation across tissues	CD44^hi^CD62L^lo^ effector-memory	CXCR3, CXCR4, CXCR6, CCR9	IL-12R, IL-18R, IL-7R, IL-15R, IL-23R	*NKT1* – IFN-γ, *NKT2* – IL-4, *NKT17* – IL-17, *NKT10* – IL-10	*NKT1* – PLZF^lo^T-bet^+^, *NKT2* – PLZF^hi^T-bet^−^RORγt^−^, *NKT17* - PLZF^int^ RORγt^+^, *NKT10* – E4BP4^+^, *NKT_FH_* – Bcl-6^+^, *iNKT_reg_* – FoxP3^+^

**Reference**	(12, 22, 48–54)	(17, 21, 22, 55, 56)	(7, 12, 13, 57–59)	(12, 15, 19, 43, 60–64)	(14, 16, 62, 65–69)	(14, 19, 65, 70–72)	(14, 17, 19, 70, 73–83)	(14, 17, 19, 50, 62, 84–90)	(14, 19, 33, 86, 91–97)

DN - double-negative/CD4^−^CD8^−^

### Phenotype

In humans, MAIT cells are predominantly CD8^+^ (70–90%), with some CD4^−^CD8^−^ (DN) (10–20%), and a minor population of CD4^+^ cells (Table 1) (12, 16, 43). iNKT cells can also be CD8^+^ (absent in mice), DN, or CD4^+^ (Table 1) (60–62, 98, 99). Within the CD8-expressing subset, both MAIT cells and iNKT cells predominantly express CD8αα homodimers or are CD8α^+^β^low^ (Table 1) (12, 61–63), in contrast to conventional T cell populations that are mainly CD8αβ^+^ (>90%). CD8αα homodimers might function to inhibit T cell activation, although their physiological role remains poorly defined (100).

Human MAIT cells display an effector-memory phenotype and characteristic expression of several surface molecules (CD161, CD26), cytokine and chemokine receptors (IL-18Rα, CCR5, CCR6), and transcription factors (PLZF, RORγt, T-bet) (Table 1) (11). As their phenotype is largely homogeneous and MR1 tetramers have only recently been developed, human MAIT cells are routinely identified using surrogate markers, most commonly as Vα7.2^+^CD161^hi^ T cells, but also using Vα7.2 combined with IL-18Rα or CD26. In contrast to the homogeneity of MAIT cells, iNKT cells show considerable heterogeneity and thus must be directly identified using CD1d/α-galactosylceramide (αGalCer) tetramers or with an antibody to the invariant Vα24-Jα18 TCRα chain in humans. While CD4^+^ and CD4^−^ iNKT cell populations show disparate expression of memory markers, chemokine receptors, and natural killer (NK) cell receptors (62, 65, 84), the predominant CD4^−^ population shows resemblance to MAIT cells, displaying an effector-memory phenotype and similar expression of surface receptors (Table 1) (65, 84, 101). Human MAIT cells coexpress the transcription factors PLZF, T-bet, and RORγt (91, 102), whereas their expression is subset specific for mouse MAIT cells, with cells expressing PLZF and either T-bet or RORγt (Table 1) (19, 102). This dichotomous expression of T-bet and RORγt is also seen in mouse iNKT cells (Table 1) (86). Therefore, the expression of a mixed Th1/Th17 transcription factor profile appears unique to human MAIT cells.

In summary, MAIT cells and iNKT cells show many overlapping characteristics, including expression of semi-invariant TCRs, recognition of non-peptide ligands, and an innate-like effector-memory phenotype. However, the phenotype of iNKT cells is considerably more heterogeneous than that of MAIT cells. MAIT cells and iNKT cells predominantly localize to peripheral tissues under homeostatic conditions, especially the liver and mucosal tissues, and are therefore optimally positioned to act as a first line of defense at the site of microbial infection.

## Mouse Models

### TCR Transgenic

Transgenic mouse models are widely used to study the phenotype and function of MAIT cells and iNKT cells, and their role in different disease settings. While use of these models has provided major contributions to our understanding of both cell subsets, it is also important to be aware of their limitations.

Mice that constitutively express the MAIT and iNKT cell invariant TCRα chain, namely, Vα19-Jα33 (termed iVα19 in several studies) (16, 103, 104) and Vα14-Jα18 (Vα14-Jα281 nomenclature used in early studies) (105, 106), respectively, have been generated on a Cα^−/−^ background. As intended, these mice have an increased frequency of the target cell population. However, as is commonly observed in TCRα transgenic models, normal T cell development is dysregulated. TCR diversity is greatly reduced, T cell numbers are significantly decreased in the thymus and many peripheral tissues, and the mice harbor an expanded population of DN T cells. In addition, as mice overexpressing the MAIT or iNKT invariant TCRα chains also harbor other T cell populations (16, 103–105), comparison of mice deficient and sufficient in MR1 or CD1d, respectively, is necessary in order to identify features specific to the cell subset of interest.

Along with global changes in T cell development, MAIT cells and iNKT cells from TCRα transgenic mice exhibit certain differences in their phenotype, function, subset distribution, and tissue localization compared with their wild-type counterparts. For example, MAIT cells from iVα19 TCRα transgenic mice display a naïve phenotype, lack expression of PLZF, and secrete considerable amounts of IL-10 and Th2 cytokines (16, 103, 104), in contrast with MAIT cells from wild-type mice (19). Moreover, while wild-type iNKT cells produce both IL-4 and IFN-γ, T cells from Vα14-Jα18 TCRα transgenic mice produce high levels of IL-4, but little IFN-γ following stimulation with αCD3 (105). However, several groups have generated refined Vα14-Jα18 TCRα mouse models using somatic cell nuclear transfer (107) or induced pluripotent stem cells (108), in which iNKT cells can secrete high levels of both IL-4 and IFN-γ.

Vβ transgenic mice, for example, Vβ6 and Vβ8 transgenic mice, can be studied as an alternative to TCRα transgenics or can be crossed with TCRα transgenics to further increase MAIT or iNKT cell frequency, and to decrease unwanted TCR specificities (16, 109). An important limitation of these models is that, as MAIT and iNKT cell populations utilize multiple TCRβ chains, the forced usage of a single Vβ will bias the antigen specificity, and thereby the functionality of the generated population. In addition to the use of double transgenics, MAIT or iNKT cell frequency can be increased by studying transgenic mice on a RAG^−/−^ or TAP^−/−^Ii^−/−^ background (16, 109). However, in these mice, interactions between MAIT or iNKT cells, and other conventional T cells (and B cells in RAG^−/−^ mice), which might influence their phenotype and development in a wild-type setting, are completely absent.

### Non-Transgenic

Given the scarcity of MAIT cells in mice and the limitations of TCR transgenic models, alternative models with increased MAIT cell frequency have been developed. A mouse strain (CAST/EiJ) with 20-fold greater frequency of MAIT cells than C57BL/6 mice was identified, and crossing these strains generated a B6-MAIT^CAST^ strain with increased frequencies of MAIT cells (35). These MAIT^CAST^ cells display a phenotype more consistent with MAIT cells from wild-type animals, including expression of PLZF, but some phenotypic and functional abnormalities remain. An alternate, non-genetic approach to increase the frequency of MAIT cells in mice is through the intranasal administration of MR1 ligand (5-OP-RU) combined with a toll-like receptor (TLR) agonist, which increases their frequency to approximately 50% of lung αβ T cells (32). Further work will be required to understand how this “boosting” may impact on the phenotype and function of MAIT cells, and thereby to establish the robustness of this experimental approach. Regardless of potential current shortcomings, efforts to develop mouse models with increased MAIT cell frequencies, while avoiding the limitations of TCR transgenic systems, appear promising.

### MHC/TCR Knockout

Models with reduced, rather than increased, MAIT or iNKT cell frequencies have also been generated either by altering the TCR repertoire or by removing the MHC molecules that are essential for MAIT or iNKT cell selection. iNKT cell-deficient Jα18^−/−^ mice are widely used; however, a recent study showed that TCRα rearrangement is perturbed in the original Jα18^−/−^ strain (110, 111). TCRα rearrangements using Jα segments upstream of Jα18 are almost completely absent, and therefore, along with other T cell populations, MAIT cell frequency is reduced. Consequently, lack of MAIT cells may contribute to the phenotype of Jα18^−/−^ mice. However, newer Jα18^−/−^ models have now been generated that exhibit a normal TCRα repertoire (except for the lack of Jα18) (112–115), thus addressing this concern.

Mice lacking MR1 or CD1d lack MAIT cells (22) or iNKT cells (116–118), respectively. However, they also lack other MR1- or CD1d-restricted T cells. A population of MR1-restricted non-MAIT T cells was recently identified in humans (119), which if present in mice would be absent in MR1^−/−^ animals. CD1d^−/−^ mice lack not only iNKT cells (type I) but also diverse (type II) NKT cells. In addition, CD1d^−/−^ mice on the BALB/c background, and to a lesser extent the C57BL/6 background, exhibit a marked increase in the frequency of MAIT cells, which might further confound studies using these mice (102). Jα18^−/−^ mice have the advantage that they lack only iNKT cells. It is important to bear this in mind when using MR1^−/−^ and CD1d^−/−^ mice, as any identified phenotypes may not be directly attributable to MAIT or iNKT cells, respectively.

Therefore, while transgenic mouse models enable the role of MAIT cells and iNKT cells to be interrogated *in vivo* in health and disease, caution is necessary when using these models. Newer models continue to be developed that aim to overcome some of the drawbacks of existing models. Nevertheless, results from any mouse model should be validated in other models to avoid findings that result from peculiarities of the chosen experimental system. Moreover, it is important to bear in mind that discoveries from mouse studies may not directly translate to humans, given the vastly different frequencies of MAIT and iNKT cells in these species, in addition to other differences, for example, in functional subsets and tissue distribution.

## Development

### Thymic Development

#### Selection

The earliest stages of MAIT cell development in the thymus show many similarities to the thymic development of iNKT cells. As with conventional T cells, the semi-invariant TCR of MAIT cells is generated *via* random recombination (49, 120); however, its formation requires an extended CD4^+^CD8^+^ (DP) thymocyte lifespan. Initial rearrangement of the TCRα locus utilizes 3′ Vα and 5′ Jα segments, with later rearrangements using progressively more 5′ Vα segments and more 3′ Jα segments (termed proximal to distal rearrangement) (121, 122). Thus, formation of the MAIT cell semi-invariant TCR that incorporates the 5′ most Vα segment (TRAV1-2) occurs late in the lifespan of DP thymocytes. A long DP thymocyte half-life is likewise necessary for generation of the iNKT TCR and hence for iNKT cell development. iNKT cells are absent in *Rorc*^−/−^ mice (RORγt-deficient) that show a reduced DP thymocyte lifespan, but their development is rescued upon expression of the rearranged Vα14-Jα18 TCRα chain or the anti-apoptotic protein Bcl-xL (123, 124). In peripheral blood T cells from *RORC*^−/−^ patients, 5′ Vα-3′ Jα TCRα pairings are absent, and hence these patients lack both MAIT cells and iNKT cells (125), presumably due to lack of rearrangement of their characteristic TCRα chains at the DP thymocyte stage.

**Figure 1:**
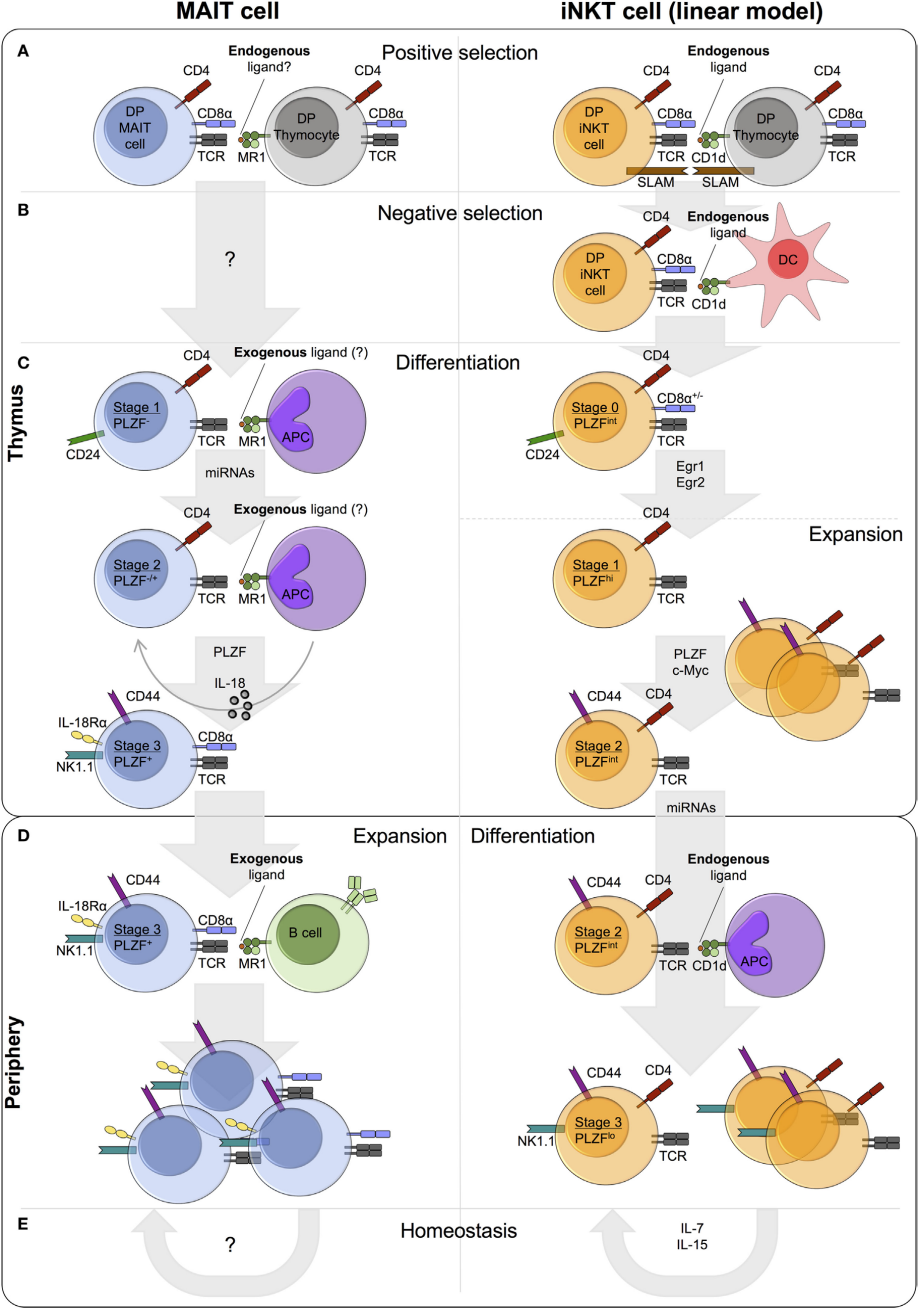
Comparison of mucosal-associated invariant T (MAIT) cell and invariant natural killer T (iNKT) cell thymic and peripheral development in mice. **(A)** MAIT cells and iNKT cells are positively selected by MR1- and CD1d-expressing DP (double-positive/CD4^+^CD8^+^) thymocytes, respectively. iNKT cell positive selection involves an endogenous ligand(s). A similar role for endogenous ligand(s) in MAIT cell selection is postulated, but such ligands have yet to be identified. Concomitant with T cell receptor (TCR)-MHC-Ib/ligand binding, homotypic interactions between signaling lymphocyte activation molecule (SLAM) family receptors are essential for iNKT cell, but not MAIT cell, development. **(B)** iNKT cells also undergo negative selection, while negative selection has not been studied for MAIT cells. **(C)** Following selection, MAIT and iNKT thymocytes differentiate through similar stages defined by the expression of CD24 and CD44. Stage 1 and stage 2 iNKT thymocytes are highly proliferative, whereas the proliferative capacity of thymic MAIT cells is currently unknown. A number of shared factors are required for thymic differentiation, including microRNAs (miRNAs) and PLZF, but the requirement for IL-18 and exogenous ligand (from commensal bacteria) is specific to MAIT cells. Conversely, the transcription factors Egr1, Egr2, and c-Myc have been implicated in iNKT cell development, but not investigated in MAIT cell development. iNKT cells express PLZF and exhibit effector functions at stage 1, while MAIT cells acquire effector capacity at stage 3. **(D)** MAIT cells and iNKT cells exit the thymus with a CD24^−^CD44^+^ memory phenotype. In the periphery, MAIT cells undergo expansion, probably driven by the presentation of exogenous ligands from commensal bacteria, whereas iNKT cell frequency remains relatively constant. **(E)** iNKT cell homeostasis is predominantly regulated by cytokines, in particular IL-7 and IL-15. By contrast, the role of MR1 and cytokines in MAIT cell homeostasis is currently unknown.

Following TCR expression, conventional T cells undergo positive selection on cortical thymic epithelial cells that present self-peptides on MHC Class I and MHC Class II. By contrast, MAIT cells are selected by MR1-expressing DP thymocytes (126, 127), comparable to the CD1d-dependent selection of iNKT cells (128, 129) (Figure 1A). For iNKT cells, selection is dependent on the presentation of endogenous lipid antigens by CD1d (Figure 1A) (130). Based on this paradigm and circumstantial evidence (131, 132), it is highly plausible that MAIT cell selection also involves an endogenous ligand(s), although such ligands have yet to be identified.

Conventional T cells are positively selected in the thymus when their TCR exhibits moderate affinity for MHC/self-peptide, while thymocytes expressing high affinity TCRs are removed from the repertoire. The strength of the TCR-MR1/ligand interaction required for MAIT cell positive selection has not been investigated, but agonist selection is hypothesized based on the following information. First, a number of unconventional T cell lineages are selected by agonist ligands, including iNKT cells, regulatory T cells, and CD8αα gut intraepithelial T cells (133). Second, compared with conventional thymocytes, the high avidity interaction between the iNKT TCR and selecting glycolipids results in strong TCR signaling (134) and therefore prolonged upregulation of the TCR-induced transcription factors Egr1 and Egr2 (135). The transcription factor PLZF, encoded by *Zbtb16*, is a direct downstream target of Egr2 (135), and both MAIT cells and iNKT cells upregulate PLZF expression during thymic development, contrasting to conventional T cells. Finally, mouse MAIT cells upregulate CD44 expression, and mouse and human MAIT cells can acquire effector function within the thymus (102), properties of antigen-experienced conventional T cells.

MR1 is essential for MAIT cell positive selection (Figure 1A) (126). However, whether engagement of other cell surface receptors is required, is currently unknown. By contrast, homotypic interactions between at least two signaling lymphocyte activation molecule (SLAM) family members (SLAMF1 and SLAMF6) are required alongside TCR-CD1d/ligand engagement, for iNKT cell development (Figure 1A). In mixed bone marrow chimeras, the frequency of iNKT cells in *Slamf1*/*Slamf6*-deficient populations is significantly reduced compared with wild-type, with a specific defect at the transition from stage 0 (CD24^hi^) to stage 1 (CD24^lo^) (136). SLAMF6 costimulation has been shown to augment TCR signaling, resulting in increased Egr2 expression and consequently enhanced expression of PLZF (137). MAIT cell development is independent of SLAM receptors, as patients deficient in SLAM-associated protein, an intracellular adaptor required for SLAM signaling, lack iNKT cells but show normal numbers of MAIT cells (16).

The role of negative selection in MAIT cell development has not been investigated (Figure 1B). By contrast, although not explicitly demonstrated, highly autoreactive iNKT cells likely undergo negative selection on DCs (Figure 1B) (138, 139). Addition of the agonist glycolipid αGalCer or CD1d overexpression during iNKT cell development results in decreased iNKT cell frequency *in vitro* and *in vivo* (138, 139). It seems likely that high avidity self-reactive MAIT thymocytes also undergo negative selection. Alternatively, peripheral MAIT cell activation could be controlled by other mechanisms, for example, dampened TCR signaling compared with conventional T cells (85).

#### Differentiation

The differentiation of thymic MAIT cells following selection remains relatively unexplored. However, a recent paper by Koay et al. identified three stages of MAIT cell development in mice and humans (102). The described developmental pathway in mice, with stages defined by the expression of CD24 and CD44 (stage 1 – CD24^+^CD44^−^, stage 2 – CD24^−^CD44^−^, stage 3 – CD24^−^CD44^+^), is remarkably similar to the linear differentiation model of iNKT cell development (Figure 1C) (130). In mice, thymic stage 1 (CD24^lo^CD44^lo^) and stage 2 (CD24^lo^CD44^hi^) iNKT cells are highly proliferative (Figure 1C) (140). Intrathymic iNKT cell proliferation requires expression of the transcription factor c-Myc (141). Mouse MAIT cells accumulate in the thymus with age, and stage 3 MAIT cells are more abundant than stage 1 and stage 2 in fetal thymic organ culture (102). These data suggest that murine MAIT cells proliferate in the thymus, similar to iNKT cells, but direct measures of *in vivo* proliferation have not been performed. By contrast, human thymic MAIT cells are present at low frequency irrespective of age (16, 102, 127), and T cell receptor excision circle (TREC) analysis of MAIT cells in human thymus and cord blood identified no differences in TREC concentration compared with conventional T cells (127). Whether human thymic and cord blood iNKT cells show enhanced proliferative capacity relative to conventional T cells is unclear, as prior studies have reported conflicting findings (73, 74). Thus, additional independent direct assessments of MAIT and iNKT thymocyte proliferation are needed to clarify the extent of their intrathymic proliferative capacity.

Development of MAIT cells along the linear developmental pathway requires a number of different factors, some of which are also necessary for iNKT cell development (Figure 1C). MR1 is essential at all stages of MAIT cell development *in vitro*, and peripheral MAIT cells are nearly absent in MR1-deficient mice (16, 19, 22, 102). Likewise, CD1d is essential for the development of iNKT cells (116–118, 142). In the absence of commensal bacteria and IL-18 *in vivo*, stage 3 MAIT cells are reduced, while the frequency (but not number) of stage 1 cells is increased (22, 102). Moreover, MAIT cell development beyond stage 1 requires microRNAs (miRNAs), as the abundance of stage 2 and stage 3 MAIT cells is decreased in *Drosha*-deficient mice (102). By contrast, PLZF is necessary only for MAIT cell maturation from stage 2 to stage 3 and for their acquisition of effector function (19, 102).

Invariant natural killer T cell development similarly requires miRNAs and PLZF (33, 92, 143). Consistent with a shared developmental niche, MAIT cell frequency is markedly increased in CD1d-deficient mice on a BALB/c background, with only minor differences to wild-type on the C57BL/6 background, although the increase in both strains is statistically significant (102). By contrast, the number of iNKT cells in the spleen and thymus of MR1-deficient mice is similar to that of wild-type, perhaps due to the much lower frequency of MAIT cells compared with iNKT cells in these mouse strains (102). In addition, the frequency of MAIT cells and iNKT cells in humans is positively correlated in adult peripheral blood (43). Despite shared development needs, the absolute requirement for commensal bacteria appears unique to MAIT cells (22, 102), as iNKT cell frequency is relatively conserved in germ-free (GF) mice compared with either specific pathogen-free (SPF) mice or mice harboring a conventional microflora (144, 145).

Stage 3 mature MAIT cells in human thymus coexpress RORγt and T-bet (102). By contrast, stage 3 MAIT cells in mice comprise two subsets, namely, RORγt^+^T-bet^−^ and T-bet^+^RORγt^−^ cells (102). Analogous to MAIT cells, thymic iNKT cell subsets have not been identified in humans, while iNKT cells comprise at least three different subsets in mouse thymus, named NKT1, NKT2, and NKT17 (discussed in more detail in Section “Subsets”) (86, 146, 147). It is unclear whether RORγt^+^T-bet^−^ and T-bet^+^RORγt^−^ MAIT cells represent different developmental stages or distinct subsets derived from a shared progenitor. Recent studies suggest that iNKT cell subsets arise from a common PLZF^hi^ precursor population and represent stable lineages with distinct transcriptional and epigenetic programs (86, 146, 147). However, whether the classic developmental stages model or the newer lineage segregation model best describes iNKT cell development, remains uncertain. Moreover, the specific signals required for commitment to the different iNKT cell subsets are largely unknown, although a multitude of factors, including cytokines and transcriptional regulators, can differentially regulate NKT1, NKT2, and NKT17 development (148). It would be worth investigating whether similar factors also modulate the differentiation of thymic MAIT cell subsets.

#### Acquisition of Innate-Like Effector Function

Mucosal-associated invariant T cells can acquire innate-like effector function in the thymus and secrete cytokines upon activation (102), comparable to iNKT cells (15, 140, 149, 150). Expression of the transcription factor PLZF is necessary and sufficient to drive innate-like effector differentiation (33, 92, 151–153). In PLZF-deficient mice, MAIT and iNKT cell development is almost completely abrogated and residual cells exhibit a CD44^lo^ phenotype, reduced expression of characteristic phenotypic markers, and impaired cytokine secretion (19, 33, 92, 102). In addition to direct regulation by Egr2 (135), PLZF expression is regulated by the binding of Runx1 to a shared intronic enhancer in several innate lymphoid lineages, including iNKT cells (154). Therefore, Runx1 likely also regulates PLZF expression in MAIT cells. During thymic MAIT cell development, PLZF expression begins at stage 2 (mouse – CD24^−^CD44^−^, human – CD161^−^CD27^+^) and is highest at stage 3 (mouse – CD24^−^CD44^+^, human – CD161^+^CD27^pos-lo^) (Figure 1C) (102). By contrast, PLZF expression is induced immediately following positive selection in iNKT cells and its expression peaks in thymic stage 1 cells (CD24^lo^CD44^lo^) (Figure 1C) (33, 92, 135). Consequently, thymic stage 1 iNKT cells can secrete cytokines upon stimulation (140, 149, 150), while MAIT cells do not acquire this functionality until stage 3 of thymic differentiation (102).

Although they can acquire effector function within the thymus, MAIT cells in humans are typically thought to exit the thymus as naïve cells and acquire their effector-memory phenotype in the periphery (14, 16, 47, 63, 127). This is supported by the naïve phenotype of MAIT cells in thymus and cord blood, and their rapid acquisition of CD45RO in neonates, such that >80% of blood MAIT cells express CD45RO by 1 month of age (14, 16, 47, 63, 127). However, further studies are required to fully define exactly when and where MAIT cells acquire their effector-memory phenotype and function, given that some thymic MAIT cells express PLZF and CD45RO (16, 102, 155). Naïve stage 2 (CD161^−^CD27^+^) MAIT cells were recently shown to predominate in human thymus and were found to a lesser degree in cord blood and young blood (102). Thus, the majority of MAIT cells may exit the thymus at stage 2 and undergo further maturation in the periphery, while a small population matures to stage 3 in the thymus (102). Stage 3 mature MAIT cells (CD24^−^CD44^+^) are the main population in mouse thymus (102). Therefore, contrasting to human MAIT cells, but comparable to mouse iNKT cells, mouse MAIT cells probably exit the thymus as CD44^+^ memory cells (140). Similarly, human iNKT cells may leave the thymus as effector-memory cells, as they already display a CD45RO^+^ memory phenotype in thymus and cord blood (66, 67, 156).

In conclusion, thymic MAIT cell development shows many similarities to that of iNKT cells, including selection on DP thymocytes, development through similar stages post-selection, a shared requirement for developmental factors, and the possibility to acquire innate-like effector function in the thymus. However, the role of MAIT cell negative selection and the extent of their intrathymic proliferation have yet to be examined. While mouse iNKT cells and likely mouse MAIT cells exit the thymus as CD44^+^ effector-memory cells, human MAIT cells appear to leave the thymus as naïve cells and acquire innate-like effector function extrathymically, although the exact timing of their thymic exit needs to be clarified. The reason for such disparity between mouse and human MAIT cells in the location of effector maturation is currently unclear.

### Peripheral Development

#### Changes in Abundance

While their frequency is relatively constant in the thymus, MAIT cells undergo a large population expansion in the periphery, reminiscent of intrathymic iNKT cell expansion in mice, increasing over 100-fold from <0.01% of T cells at birth to 1–10% of T cells by adulthood (14, 41–43, 47, 102). The increase in MAIT cell numbers is gradual and occurs over a number of years, although estimates for the age at which adult frequencies are reached vary between studies (6–25 years of age) (14, 41–43, 47, 102). By contrast, peripheral iNKT cell frequencies remain relatively constant from birth to adulthood (66, 157, 158).

Though MAIT cell thymic selection is independent of B cells (16), B cells are crucial for peripheral MAIT cell expansion in mice (Figure 1D). Peripheral MAIT cells are almost entirely absent in B cell-deficient mice, and transfer of B cells is sufficient to induce MAIT cell expansion in iVα19/Vβ6 RAG^−/−^ mice (16, 22). Whether cognate interactions between B cells and MAIT cells are necessary for such expansion has not been established. In humans, the role of B cells remains uncertain. Treiner et al. observed a reduced frequency of MAIT cells (as measured by the presence of Vα7.2-Jα33 TCRα transcripts) in the blood of patients who lack B cells due to a mutation in Bruton tyrosine kinase (*BTK*) (22). However, only four patients were analyzed, one of which had a normal number of MAIT cells. A study of common variable immunodeficiency (CVID) provides indirect evidence against a role for B cells in regulating human MAIT cell frequency (159). Although the abundance of B cells and MAIT cells was variably decreased in CVID patients, the frequency of MAIT cells showed no correlation with that of B cells. A major confounding factor in these human studies is the increased occurrence of infections in patients with *BTK* deficiency and CVID, which can independently modulate MAIT cell frequency. Thus, whether B cells have a role in MAIT cell expansion or at other stages of their development in humans requires further investigation. B cells are not essential for iNKT cell development, but they do play an important role in human peripheral iNKT cell homeostasis (160), as discussed below.

It is widely hypothesized that peripheral MAIT cell expansion and maturation is driven by the presentation of microbial antigens on MR1, derived from either commensal or pathogenic bacteria (Figure 1D). Although this has yet to be formally proven, a variety of evidence supports this hypothesis. In humans, MAIT cells are naïve in thymus, cord blood, and in newborns, but rapidly acquire a memory phenotype in the blood during the first month of life (14, 16, 47, 102), concomitant with exposure to bacteria. MAIT cells are absent in GF mice (22) and expand upon microbial reconstitution with a single strain of bacteria (17). Furthermore, MAIT cells undergo MR1-dependent proliferation *in vitro* and *in vivo* in response to bacteria, for example, in the lungs of mice infected with *Salmonella enterica* serovar Typhimurium (32, 91, 161). The TCR repertoire of MAIT cells also supports microbe-mediated expansion. While the TCR repertoire is polyclonal in cord blood, it is oligoclonal in adult blood (47, 49, 50, 63), consistent with the hypothesized expansion of specific clones in response to particular bacteria. This is plausible, as MAIT cells with distinct TCRs are activated *in vitro* following stimulation with different bacteria (162), and in a human *in vivo Salmonella enterica* serovar Paratyphi A challenge setting, the relative abundance of different MAIT cell clonotypes changes in response to infection (163).

#### Phenotypic and Functional Maturation

In addition to expansion, MAIT cells undergo maturation in the periphery, as indicated by marked phenotypic changes. Approximately half of MAIT cells in the thymus are either DP or CD4^+^, whereas MAIT cells in adult blood are predominantly DN or CD8^+^ (Figure 1D) (16, 49, 102). Furthermore, CD8^+^ MAIT cells in the thymus and cord blood are CD8αβ^+^, whereas roughly half of CD8α^+^ MAIT cells in adult blood express CD8αα homodimers (16, 63, 102). CD8αα^+^ MAIT cells are thought to arise from CD161^hi^CD8αβ^+^ cells in the periphery (47, 63). iNKT cells undergo similar phenotypic changes with age. CD4^+^ cells comprise 80–90% of iNKT cells in human thymus and cord blood, and progressively decline in the periphery to comprise on average 40% of iNKT cells in adult blood (15, 73, 158). This may result from the preferential peripheral expansion of CD4^−^ iNKT cells, as CD4^−^ iNKT cells show reduced TREC content and increased proliferation in response to IL-15 compared with CD4^+^ iNKT cells (73). However, alternative explanations, such as CD4 downregulation, remain possible. It is unknown if CD4^+^ and CD8^+^ MAIT cells show differences in their proliferative capacity. Analogous to MAIT cells, a large proportion of CD8^+^ iNKT cells in human blood express CD8αα (61, 62), but whether these arise in the periphery has not been investigated.

Following thymic exit, MAIT cells acquire a memory CD45RO^+^ phenotype and upregulate the expression of characteristic phenotypic markers, such as CD161 and IL-18Rα (Figure 1D), while downregulating the expression of lymph node homing receptors, including CD62L and CCR7 (14, 47, 63, 102, 127, 155). These changes are gradual, as MAIT cells in cord blood, young blood, and adult blood exhibit an increasingly mature phenotype (47, 63, 102, 127, 155). iNKT cells undergo similar extrathymic phenotypic changes (15, 67, 73, 156, 158, 164), although they already exhibit a memory phenotype in the thymus and cord blood (15, 66, 67, 140). Upregulation of NK cell receptors on mouse iNKT cells is dependent on CD1d (142), while IL-7 can upregulate CD161 on human cord blood iNKT cells *in vitro* (74). The signals required for NK cell receptor upregulation on developing MAIT cells are currently unknown.

As well as phenotypic changes, MAIT cells undergo further functional differentiation following thymic exit. Although stage 3 MAIT cells in human thymus can produce IFN-γ and TNF-α following PMA and ionomycin stimulation, their capacity to do so is significantly decreased compared with peripheral blood MAIT cells (102). Moreover, the majority of human MAIT cells may exit the thymus at stage 2 before they acquire effector capacity. In contrast to adult blood Vα7.2^+^CD161^hi^ T cells, cord blood Vα7.2^+^CD161^hi^ T cells are unable to produce IFN-γ or Granzyme B in response to overnight stimulation with *Escherichia coli*-infected THP-1 cells (47). However, as MAIT cells comprise the majority of Vα7.2^+^CD161^hi^ T cells in adult blood, but only a small fraction of Vα7.2^+^CD161^hi^ T cells in cord blood (47), whether this finding also applies to MAIT cells needs to be confirmed using the MR1 tetramer. Similar to the findings for MAIT/Vα7.2^+^CD161^hi^ T cells, human thymic and cord blood iNKT cells appear functionally immature compared with adult blood iNKT cells. Early reports suggested that thymic and cord blood iNKT cells were incapable of cytokine production without prior *in vitro* expansion (66, 73). However, more recently, freshly isolated iNKT cells from the thymus and cord blood were shown to secrete cytokines, including IFN-γ, TNF-α, and IL-4, in response to TCR and/or PMA and ionomycin stimulation (15, 74). Consequently, the capacity of human thymic and cord blood iNKT cells to produce cytokines needs to be clarified. In contrast to human MAIT cells and iNKT cells, mouse thymic MAIT (102) and iNKT (140, 149, 150) cells strongly produce cytokines, suggesting possible species-specific differences in when cytokine-producing capacity is acquired.

#### Homeostasis

The requirements for MAIT cell proliferation and survival in the periphery are poorly understood (Figure 1E). Conventional memory T cells depend predominantly on cytokines for peripheral maintenance (165), suggesting stimulation with cytokines, as opposed to MR1, might be key for MAIT cell homeostasis. iNKT cells exhibit subset-specific requirements for cytokines. While IL-15 is indispensable for the survival and functional maturation of most iNKT cells in mice (75, 166), NKT17 cell homeostasis is exclusively dependent on IL-7 (76) (Figure 1E). Moreover, IL-15 and IL-7 preferentially stimulate the proliferation of CD4^−^ and CD4^+^ human iNKT cells, respectively (73). In contrast to iNKT cells, MAIT cells proliferate only in response to IL-15 (161), and not IL-7 (91), despite their exquisite sensitivity to stimulation with either cytokine (30, 91, 167–169). Cytokines that signal *via* STAT3 are required for human MAIT and iNKT cell development and homeostasis, as indicated by the 4- and 20-fold reduction in their frequency, respectively, in patients with heterozygous loss-of-function mutations in *STAT3* (77). The central role of STAT3 appears to be downstream of the IL-23 receptor (and possibly the IL-21 receptor) in MAIT cells, and the IL-21 receptor in iNKT cells. IL-18 is similarly necessary for MAIT cell development and/or survival, as IL-18-deficient mice exhibit reduced thymic and peripheral MAIT cell frequencies (102). Interestingly, the role of IL-18 appears independent of IL-18 receptor signaling, as MAIT cell development is normal in IL-18Rα-deficient mice (102). Therefore, further work is necessary to determine the specific role of IL-23 and IL-18 in regulating MAIT cell frequency and to establish the requirement for IL-7, IL-15, and additional cytokines in MAIT cell homeostasis. Furthermore, it remains to be investigated whether RORγt^+^ MAIT cells and T-bet^+^ MAIT cells in mice are differentially regulated by cytokines, as has been demonstrated for the equivalent murine iNKT cell subsets.

It is unknown if tonic TCR signaling is necessary for MAIT cell homeostasis (Figure 1E). iNKT cell homeostasis in mice appears independent of CD1d (Figure 1E). iNKT cells can survive for weeks in the periphery of mice in the absence of CD1d (142, 170), and the homeostatic expansion of iNKT cells in lymphopenic hosts is CD1d independent (75, 166). By contrast, CD1d may play a role in human iNKT cell homeostasis through lipid antigen presentation on B cells. Compared with iNKT cells from total PBMCs, iNKT cells from B cell-depleted PBMCs (but not from PBMCs depleted of other CD1d^+^ populations) display reduced proliferation and cytokine production *in vitro* upon stimulation with αGalCer + IL-2 (160). In addition, iNKT cells exhibit decreased frequency and altered functionality in systemic lupus erythematosus patients, associated with reduced CD1d expression on immature B cells (160, 171, 172). Restoration of CD1d expression is sufficient to reverse these defects both *in vitro* and *in vivo* (160). Thus, it is worth examining whether MR1 has a role in MAIT cell homeostasis, particularly in humans. However, although MR1 is widely expressed by hematopoietic and non-hematopoietic cells, it is largely retained in the endoplasmic reticulum prior to ligand exposure (132, 173–175). Consequently, the ability of MR1 to modulate MAIT cell homeostasis may be limited compared with CD1d, which is frequently present at the cell surface (176).

In conclusion, MAIT cells and iNKT cells undergo further extrathymic maturation. However, while peripheral iNKT cell frequency remains relatively constant with age, MAIT cells undergo a large population expansion from birth to adulthood. B cells have an important, but differing, role in MAIT cell and iNKT cell peripheral development. Compared with MAIT cells, more is known about the role of cytokines in the peripheral maintenance of iNKT cells. Given that MAIT cells express similar cytokine receptors to iNKT cells, including the receptors for IL-7 and IL-15 (14), it is worth investigating the role of these cytokines in MAIT cell homeostasis and peripheral maturation. With the availability of MR1 tetramers (12, 13) and mice with an increased frequency of MAIT cells (35), this can now be examined *in vivo* using cytokine-deficient mice.

### Fetal Development

Similar to human iNKT cells (64), human MAIT cells develop in fetal thymus and can be identified in both lymphoid and non-lymphoid peripheral tissues in the second trimester of fetal development (155). As the timing of early MAIT cell and iNKT cell development in humans is comparable (47, 64, 155), and iNKT cells develop postnatally in mouse thymus (150, 177), it is likely that MAIT cells also undergo postnatal development in mice.

Before discussing human fetal MAIT cell development, it is important to note that, while fetal iNKT cell development has been studied using the CD1d tetramer, fetal MAIT cell development has so far only been investigated using the surrogate MAIT cell markers Vα7.2 and CD161. As previously mentioned, the MAIT cell populations defined as MR1/5-OP-RU tetramer^+^ or Vα7.2^+^CD161^hi^ are essentially the same in adult blood, while MR1/5-OP-RU tetramer^+^ MAIT cells comprise <20% of Vα7.2^+^CD161^hi^ T cells at birth (47). Moreover, the majority of MAIT cells in human thymus are CD161^−^CD27^+^ stage 2 cells, and stage 2 MAIT cells are also present at lower frequencies in cord blood and young blood (~20% and ~10% of MR1/5-OP-RU tetramer^+^ MAIT cells, respectively) (102). Therefore, using Vα7.2 and CD161 for fetal MAIT cell identification will fail to capture these CD161^−^ MR1/5-OP-RU tetramer^+^ MAIT cells. Overall, findings from the study of Vα7.2^+^CD161^hi^ T cell development in fetal tissues may not accurately reflect the developmental pathway of MAIT cells. This should be taken into consideration when interpreting the findings discussed below, all of which were made using Vα7.2 and CD161 to identify “MAIT” cells.

#### Frequency and Localization

During fetal development, Vα7.2^+^CD161^hi^ T cells comprise ~0.05% of T cells in human thymus, significantly lower than their frequency in adult blood (155). Their frequency in the thymus remains low and relatively constant after birth, at least up until the age of 14 (102). In contrast to MAIT cells, iNKT cell frequency in early fetal thymus is similar to that in adult blood (~0.1% of T cells) (156). However, their frequency decreases with gestational age in the thymus, cord blood, and neonatal peripheral blood, such that they are rare in postnatal thymus, while it increases in fetal peripheral tissues, particularly the small intestine and spleen (47, 64, 73, 156, 158, 178). This suggests a wave of iNKT cell development in the thymus early during fetal life (156), accompanied by the gradual population of peripheral tissues. This might also be true for MAIT cells, as the frequency of Vα7.2^+^CD161^hi^ T cells in the blood of neonates decreases with gestational age (47). However, whether their thymic frequency also decreases, is currently unknown. Contrary to iNKT cells, no correlation was observed between gestational age and Vα7.2^+^CD161^hi^ T cell frequency in fetal tissues (155), although the sample size was relatively low.

As in adults, Vα7.2^+^CD161^hi^ T cells are enriched in fetal peripheral tissues, including the lung, liver, and small intestine, with lower frequencies in the thymus and secondary lymphoid organs (SLOs) (155). iNKT cells are similarly enriched in the small intestine, but relatively depleted in the liver, lung, and SLOs (64). The frequency of Vα7.2^+^CD161^hi^ T cells in fetal tissues is low compared with the corresponding adult tissues, particularly in the liver, where they are ~100-fold less frequent in the fetus (14, 155). The frequency of human iNKT cells in adult peripheral tissues is poorly characterized, due to their low abundance. Nonetheless, at least in liver and spleen (15, 179, 180), their frequency appears largely similar to that in fetal tissues.

#### Phenotypic and Functional Maturation

In all fetal tissues, Vα7.2^+^CD161^hi^ T cells are less differentiated than in adult blood (155), analogous to iNKT cells (64, 74, 156). Nevertheless, Vα7.2^+^CD161^hi^ T cells in fetal peripheral tissues, particularly the small intestine, show a more mature phenotype than those in the thymus and SLOs, with increased expression of IL-18Rα and CD45RO, and reduced expression of CD62L (155). In addition, peripheral tissue Vα7.2^+^CD161^hi^ T cells are functionally more mature than their counterparts in lymphoid organs, producing increased IFN-γ *in vitro* following *E. coli* stimulation (155). Similarly, iNKT cells are phenotypically and functionally more mature in peripheral tissues compared with lymphoid organs (64). However, while Vα7.2^+^CD161^hi^ T cells are naïve in cord blood (16) and only a fraction express CD45RO in fetal thymus (155), >80% of iNKT cells exhibit a memory CD45RO^+^ phenotype in both cord blood (66, 67) and fetal thymus (156). Moreover, the proportion of Vα7.2^+^CD161^hi^ T cells that produce IFN-γ is significantly reduced in fetal peripheral tissues compared with adult blood (155), whereas iNKT cells in fetal small intestine and adult blood show largely comparable IFN-γ production in response to αGalCer (64). As iNKT cells from GF mice exhibit reduced cytokine production compared with their counterparts from standard SPF mice (181), and the fetal environment is typically thought to be sterile (182), it is perhaps surprising that fetal iNKT cells do not display reduced functionality compared with those in adult peripheral blood. However, this could be understood if the fetal environment was not entirely sterile, conflicting with the “sterile womb hypothesis” (182). In support of this suggestion, a number of recent papers provide evidence for the presence of microbes during fetal development, although these findings remain highly controversial [reviewed in Ref. (182)].

The discovery of mature CD45RO^+^ Vα7.2^+^CD161^hi^ T cells in fetal peripheral tissues appears at odds with the requirement for commensal bacteria for the development and maturation of MAIT cells in mice (17, 22, 102). Moreover, MAIT cells exhibit a naïve CD45RA^+^ phenotype in cord blood (16) and neonates (47), and rapidly upregulate CD45RO following birth, concomitant with their exposure to riboflavin-synthesizing commensal bacteria (14, 16, 47, 102). This suggests microbe-driven maturation of human MAIT cells, akin to mouse MAIT cells. The reason for the discordant findings in fetal tissues and postnatal blood is currently unclear. As mentioned earlier, it is possible that MAIT cells in peripheral tissues comprise largely tissue-resident populations distinct from those in blood, similar to what has been proposed for iNKT cells based on parabiosis experiments in mice (28, 29). However, this has yet to be investigated for MAIT cells. Regardless, this would not explain why MAIT cells undergo maturation in fetal peripheral tissues. Maturation could be understood if the fetal environment was not completely GF, as discussed above. Alternatively, it is possible that other unknown factors can mediate fetal MAIT cell maturation.

In summary, we have a very limited understanding of fetal MAIT cell development. Only one paper has addressed MAIT cell development in human fetal tissues and the MR1 tetramer was not used in this study. Nevertheless, the findings for Vα7.2^+^CD161^hi^ T cells are reminiscent of iNKT cell fetal development, with Vα7.2^+^CD161^hi^ T cells undergoing maturation in fetal peripheral tissues, particularly at mucosal sites. Now that MR1 tetramers are readily available, it will be necessary to establish whether MR1/5-OP-RU tetramer^+^ MAIT cells show similar fetal maturation to Vα7.2^+^CD161^hi^ T cells and if so, to explore the factors driving such maturation.

## Activation

### Mechanisms

**Figure 2:**
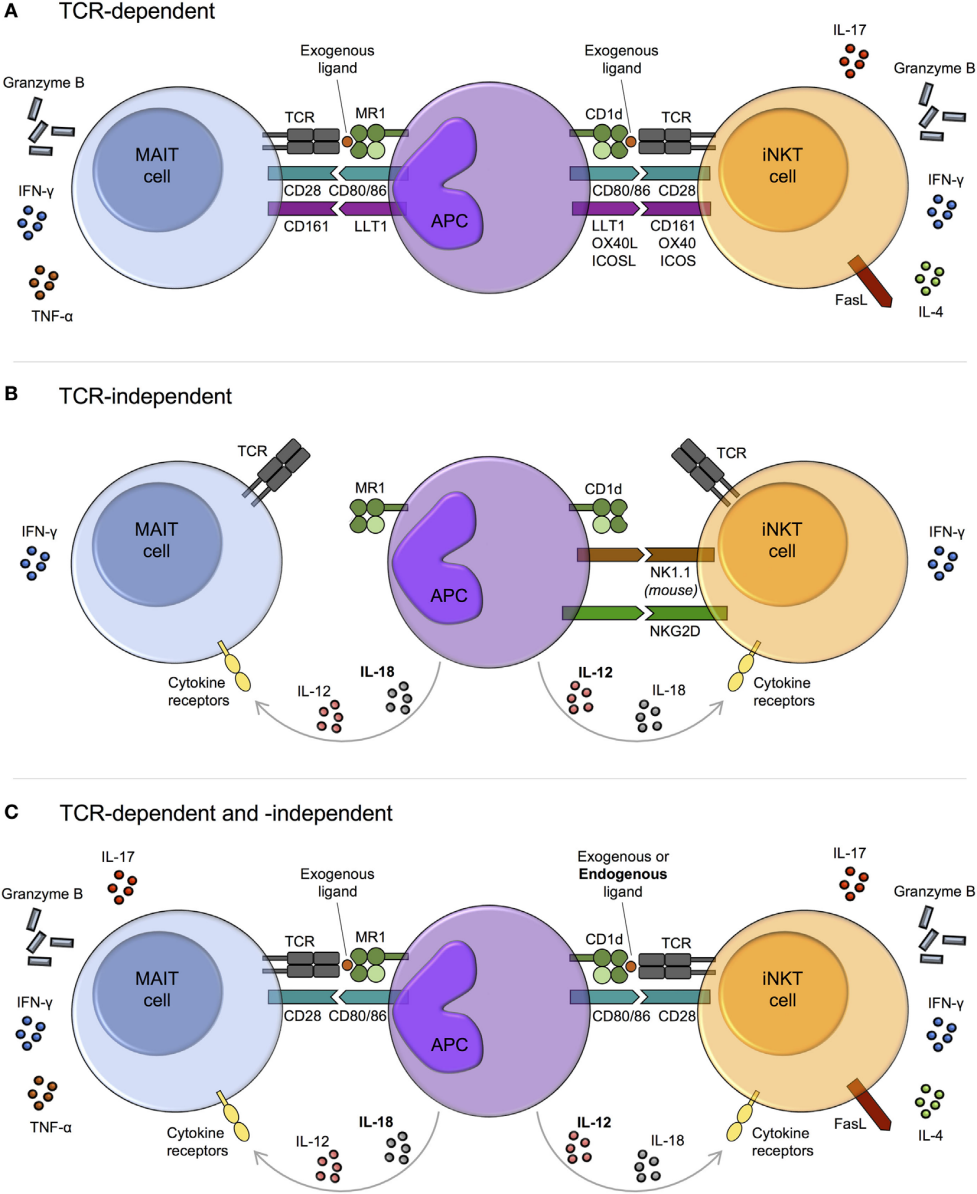
Mechanisms of mucosal-associated invariant T (MAIT) cell and invariant natural killer T (iNKT) cell activation. MAIT cells and iNKT cells are activated *via* three main pathways: T cell receptor (TCR)-dependent **(A)**, TCR-independent (predominantly cytokine-driven) **(B)**, and combined TCR-dependent and -independent **(C)**. **(A)** In response to TCR signaling, MAIT cells and iNKT cells produce cytokines and exhibit cytotoxic activity. The degree of activation is modulated by costimulatory molecules, including CD161. The array of cytokines produced by iNKT cells varies upon activation of different costimulatory pathways. Whether this is also the case for MAIT cells is currently unknown. **(B)** TCR-independent activation is largely cytokine-mediated, with similar combinations of cytokines capable of activating both MAIT cells and iNKT cells, for example, IL-12 + IL-18. However, while IL-12 (bold) appears dominant for iNKT cell activation, IL-18 (bold) is key for MAIT cells. Cytokine-dependent activation of MAIT and iNKT cells may require prior TCR stimulation, as has been reported for human iNKT cells. As well as by cytokines, iNKT cells can be directly activated *via* certain natural killer (NK) cell receptors, such as NK1.1 in mice. IFN-γ production may predominate following TCR-independent activation, although this requires further investigation. **(C)** MAIT cells and iNKT cells can be activated through a combination of TCR and cytokine signaling. In this setting, iNKT cell antigens are typically weak self-antigens (bold), but endogenous ligands for MAIT cells have not been identified.

Analogous to iNKT cells, MAIT cells can be activated by TCR signals, cytokine signals independent of the TCR, or by combined TCR and cytokine signals (Figure 2).

The MAIT cell semi-invariant TCR recognizes bacterial and yeast riboflavin metabolite ligands in the context of MR1, with the most potent ligands being 5-OP-RU and 5-OE-RU [5-(2-oxoethylideneamino)-6-d-ribitylaminouracil] (Table 1; Figure 2A) (7, 13). By contrast, iNKT cells recognize various glycolipid and phospholipid antigens bound to CD1d (Table 1; Figure 2A), including glycosphingolipids from *Sphingomonas* spp. and *Bacteroides fragilis*, diacylglycerols from *Borrelia burgdorferi* and *Streptococcus pneumoniae*, and endogenous lysophospholipids (57). Although a wide range of lipid antigens have been identified for iNKT cells, compared with only a few for MAIT cells, the list of known antigens for both subsets is likely not exhaustive.

As the riboflavin synthesis pathway is present in diverse pathogenic and commensal bacteria, as well as in yeast, but absent in mammals, recognition of riboflavin metabolites enables MAIT cells to effectively discriminate self from non-self. No endogenous ligands have been identified for MAIT cells, although this is an active area of research. The ability of bacteria to activate MAIT cells *in vitro* strongly correlates with the presence of the riboflavin metabolic pathway (7) and activation of MAIT cells by a number of viruses, including dengue, influenza A, and hepatitis C, is MR1-independent (168, 183–185). Thus, presentation of endogenous ligands by MR1 does not appear to be important for MAIT cell activation in bacterial or viral infections. However, endogenous ligands could have a key role *in vivo* in inflammation and cancer, or in specific infectious settings. This is plausible, as MR1 can bind endogenous ligands to activate non-MAIT T cells (119). In contrast with MAIT cells, self-lipid ligands are known to play a key role in iNKT cell biology. Although a number of endogenous ligands can activate iNKT cells, including lysophosphatidylcholine (human iNKT cells only) and ether-bonded mono-alkyl glycerophosphates (186, 187), recent evidence suggests that, at least in mice, α-linked glycosylceramides are the major endogenous ligands (188).

In the absence of riboflavin metabolites, MAIT cells can be activated by cytokines independent of their TCR (Figure 2B). Similar to iNKT cells (189–192), they are potently activated by IL-12 + IL-18, as well as by various combinations of IL-12, IL-15, IL-18, and type I interferons (30, 167, 168, 193). In general, a single cytokine is insufficient to induce significant activation. MAIT cells express high levels of IL-18Rα and IL-18 appears dominant for their TCR-independent activation, at least in viral infections (Figure 2B) (17, 168, 183). By contrast, IL-12 is key for iNKT cell activation in the absence of TCR stimulation (Figure 2B) (78, 191, 192).

It is unknown if MAIT cells are permanently amenable to TCR-independent cytokine stimulation. The capacity of human iNKT cells to respond to cytokine stimulation alone appears to reflect a transitory state that depends on prior TCR stimulation. In response to weak TCR stimulation by CD1d/self-lipid, histone H4 acetylation at the *IFNG* locus leads to a transient increase in the responsiveness of iNKT cells to innate stimulation with IL-12 + IL-18 independent of additional TCR signaling, which decays over a period of hours to days (79). As iNKT cells adoptively transferred into CD1d^−/−^ or CD1d^+/+^ mice show comparable responses to a number of bacteria and viruses (191, 194), cytokine-dependent activation of mouse iNKT cells may be entirely TCR independent. However, it remains possible that iNKT cells undergo TCR signaling in donor mice prior to adoptive transfer.

Mucosal-associated invariant T cells can integrate both TCR- and cytokine-dependent signals to augment functional capacity (30, 91, 167, 169, 193, 195, 196), similar to iNKT cells (197–199) (Figure 2C). Many of the cytokines that can drive TCR-independent activation have been shown to costimulate TCR signaling for MAIT cells, including IL-12, IL-15, and/or IL-18. These are typically produced by antigen-presenting cells (APCs) downstream of TLR activation. MAIT cell activation following *E. coli* stimulation of THP-1 cells is mediated by TLR4-induced IL-12 + IL-18, combined with MR1-dependent TCR activation by microbial ligand(s) (193). In this model, early MAIT cell activation depends predominantly on TCR signals, while both TCR and cytokine signals are crucial at later time points. In a similar manner, iNKT cells are activated by self-lipids together with IL-12 following TLR4 or TLR7/8 stimulation of DCs (197, 198, 200). The mechanisms underlying TCR and cytokine synergy in MAIT cells and iNKT cells remain to be established. However, TCR signaling-induced histone acetylation at the *IFNG* locus (79), as discussed above, may play a role in iNKT cells.

As MAIT cells are hyporesponsive to stimulation *via* the TCR alone, synergy between TCR and cytokine signaling likely plays a key role in robust MAIT cell activation *in vivo* (30, 85, 169). This is supported by a recent study showing that both metabolites from the riboflavin biosynthesis pathway and costimulatory signals are required for MAIT cell accumulation *in vivo* following bacterial lung infection (32). For iNKT cells, innate signaling from IL-12 provides the dominant signal for activation in many bacterial infections, even in the presence of cognate microbial lipid antigens (78). Cytokine signaling might also dominate in activating MAIT cells. TCR stimulation is insufficient to induce sustained MAIT cell effector responses *in vitro* (30), and in certain bacterial settings, blocking cytokines, as opposed to MR1, has a greater impact on MAIT cell activation (195, 201). Moreover, a central role for cytokines would potentially explain why MAIT cells are not constitutively activated by TCR-dependent sensing of commensal bacteria. However, the relative role of TCR- and cytokine-mediated activation will be influenced by many factors, including the nature of the APC. MAIT cell activation in response to *Streptococcus pneumoniae in vitro* is driven purely by cytokines when THP-1 cells are used as the APC, whereas in the presence of monocyte-derived macrophages, activation is driven by both MR1 and cytokines (201).

In addition to TCR- and cytokine-dependent activation, MAIT cells could potentially be activated *via* NK cell receptors, some of which can directly activate iNKT cells (Figure 2B). For example, NKG2D engagement triggers degranulation of human CD4^−^ iNKT cells (202), and mouse iNKT cells produce IFN-γ following crosslinking of NK1.1 (203), although the significance of TCR- and cytokine-independent activation *in vivo* remains unknown. In contrast to iNKT cells, direct NK cell receptor-mediated activation of MAIT cells has yet to be reported. Despite high expression of NKG2D (14), the cytotoxic activity of MAIT cells against *E. coli*-infected HeLa cells (overexpressing MR1) *in vitro* is unaffected by the presence of anti-NKG2D antibody (204). Nevertheless, reports have suggested both costimulatory and coinhibitory roles for the NK cell receptor CD161 on MAIT cells (80, 204). Similarly, CD161 can costimulate the activation of human iNKT cells (205).

#### Regulation

Costimulatory and coinhibitory molecules, including CD28, ICOS, OX40, and PD-1, have an important role in regulating iNKT cell activation and effector function *in vitro* and *in vivo* (Figure 2A) (206, 207). In addition to simply augmenting or dampening the magnitude of responses, engagement of specific costimulatory receptors on iNKT cells has been shown to skew the induced effector response (206, 207). For example, blocking the interaction of CD28 with CD86 more strongly inhibits IFN-γ production compared with IL-4 production by murine iNKT cells *in vitro* in response to αGalCer, thus promoting a Th2-biased response (208). While MAIT cells express various costimulatory and coinhibitory molecules (209), we have limited understanding of their functional role, although a few have been shown to modulate MAIT cell effector function *in vitro* (85, 210–212). For example, costimulation with αCD28 augments MAIT cell cytokine production and proliferation upon αCD3 stimulation (85). By contrast, the coinhibitory molecule PD-1 is upregulated on MAIT cells in several bacterial and viral infections, including hepatitis C (213) and tuberculosis (TB) (211), and PD-1 blockade leads to enhanced IFN-γ production by MAIT cells from active TB patients in response to *in vitro* stimulation with live bacillus Calmette–Guérin (211). Nevertheless, the role of costimulatory and coinhibitory molecules in modulating MAIT cell activation *in vivo*, and their capacity to differentially skew the MAIT cell effector response, has yet to be investigated.

In addition to the expression of coinhibitory molecules, two additional mechanisms may function to negatively regulate MAIT cell activation and/or to switch off MAIT cell effector functions upon resolution of infection or inflammation. First, MR1-binding antagonist ligands, including 6-FP (6-formylpterin) and Ac-6-FP (acetyl-6-formylpterin), competitively inhibit MAIT cell activation *in vitro* in response to synthetic agonist ligand (Table 1) (58, 59, 214). Furthermore, intranasal administration of Ac-6-FP can inhibit MAIT cell accumulation in a dose-dependent manner in the lungs of mice following intranasal administration of 5-OP-RU and a TLR agonist (riboflavin-deficient *Salmonella enterica* serovar Typhimurium) (59). Second, MAIT cells exhibit a proapoptotic phenotype driven by PLZF, akin to iNKT cells (215). This sensitivity to activation-induced cell death may function to restrain the MAIT cell effector response in order to minimize immunopathology. However, the role of these mechanisms in regulating MAIT cell activation in physiological settings is currently unknown.

#### Therapeutic Modulation

Although microbe-driven TCR-dependent MAIT cell activation requires expression of the riboflavin biosynthesis pathway (7), non-riboflavin activating and inhibitory MR1 ligands have recently been identified (59). Keller et al. used various *in silico* approaches to screen libraries of small organic molecules and drugs for potential MR1 ligands (59). Identified targets were then tested for their ability to modulate MR1 expression and MAIT cell activation. Metabolites of the drug diclofenac were found to activate certain Jurkat MAIT cell lines *in vitro*, depending on their TCRβ chain usage, whereas 3-F-SA (3-formylsalicylic acid) could inhibit 5-OP-RU-dependent MAIT cell activation *in vitro* and *in vivo* (Table 1). While these findings imply that common therapeutics might inadvertently affect human MAIT cell activity *in vivo*, they also indicate the potential to design drugs to modulate MAIT cell function. Whether iNKT cell activation and function might also be influenced by common drugs is unknown. However, as lipid-based drug delivery systems are increasingly employed to improve oral bioavailability (216), it will be important to investigate the impact of such lipid-based formulations on iNKT cell function.

To summarize, diverse activation mechanisms are available for MAIT cells that are largely shared with iNKT cells. However, whereas iNKT cells can be activated by self-ligands in combination with cytokines, endogenous ligands for the MAIT cell TCR are yet to be identified. Due to their capacity for cytokine-mediated activation, MAIT cells and iNKT cells can play key roles in diverse infectious, as well as inflammatory and malignant diseases, even in the absence of their cognate microbial antigens. The relative importance of TCR- and cytokine-dependent activation *in vivo* is likely to be context-dependent and influenced by the nature of the pathogen and its TCR/TLR ligands, the type of activated APCs, the availability of costimulatory/coinhibitory molecules, and the stage of infection or inflammation. Nevertheless, the role for cytokines appears more important than for conventional T cell activation and may even dominate in MAIT cell and iNKT cell activation in some settings, despite the presence of microbial TCR ligands.

## Effector Functions

### Cytokine Production

Upon activation, MAIT cells rapidly produce cytokines such as IFN-γ, TNF-α, and IL-17 (Table 1) (14). MAIT cells typically secrete a limited range of pro-inflammatory cytokines. By contrast, iNKT cells secrete a huge variety of both pro- and anti-inflammatory cytokines, including IL-4, IFN-γ, IL-10, and IL-17 (Table 1) (9).

The factors that govern which cytokines MAIT cells produce under different stimulatory conditions remain poorly characterized. Human MAIT cells secrete IFN-γ following both TCR- and cytokine-dependent activation, whereas TNF-α production is more contingent on TCR signaling (Figure 2) (14, 30). Though all human MAIT cells express RORγt, in addition to other type 17-associated molecules, such as CCR6 and the IL-23 receptor (14, 70), IL-17 production *ex vivo* is usually only detected following PMA and ionomycin stimulation (14, 70), and not upon TCR or cytokine stimulation alone (14, 30, 85, 169). However, certain cytokines, such as IL-7 or IL-23 + IL-1β, can induce IL-17 production when combined with a TCR stimulus (169). In addition, MAIT cells may exhibit functional plasticity driven by cytokines, as has been demonstrated *in vitro*. For example, CD161^hi^CD8α^+^ T cells (predominantly MAIT cells) develop a more Tc1-like phenotype following culture with αCD3/αCD28 + IL-12 for 14 days (85).

Similar to MAIT cells, the profile of cytokines produced by iNKT cells varies under different stimulation conditions and there is limited knowledge regarding the factors that regulate this. iNKT cells secrete both IFN-γ and IL-4 upon TCR stimulation with microbial antigens (Figure 2A) (217). By contrast, cytokine-dependent activation by viruses or TLR ligands stimulates predominantly IFN-γ production and not IL-4 (Figure 2B) (217). Chemically modified analogs of the iNKT cell ligand αGalCer have been identified that induce qualitatively different cytokine responses *in vitro* and *in vivo*, specifically Th1-biased, Th2-biased, or mixed Th1/Th2 responses (218–220). Although the exact mechanisms for this remain unknown, the stability of ternary TCR-CD1d/glycolipid complexes appears to have an important role, with prolonged TCR stimulation favoring Th1-biased responses (218, 220). As discussed, activation of different costimulatory pathways can also skew the iNKT cell response to antigen stimulation (206, 207). Therefore, the type of lipid antigens and costimulatory molecules available to activate iNKT cells *in vivo* will alter the nature of the cytokine response. Whether different MAIT cell ligands/chemical modifications of MAIT cell ligands, or costimulatory pathways, can skew MAIT cell cytokine production, remains to be investigated. As the range of cytokines produced by MAIT cells is less functionally diverse than that of iNKT cells, the capacity to drastically alter the overall immune response by skewing MAIT cell cytokine production may be more limited than with iNKT cells.

Human MAIT cells in different tissues exhibit differential cytokine production. MAIT cells in the female genital mucosa appear skewed toward type 17 functions, secreting increased IL-17 and IL-22, and decreased IFN-γ and TNF-α, compared with blood MAIT cells (26). IL-22-secreting MAIT cells are also enriched in fetal small intestine (155), while MAIT cells in adipose tissue exhibit the unique capacity to secrete IL-10 (20). Different iNKT cell subsets preferentially localize to certain tissues in mice (18, 87). As a result, challenge with αGalCer induces distinct cytokine responses depending on the route of antigen delivery and thus the nature of the iNKT cell subsets activated (18). Whether variation in MAIT cell cytokine production across tissues can similarly be explained by the tissue-specific enrichment of different MAIT cell subsets is currently unknown. Of interest, IL-10-producing NKT10 cells preferentially localize to adipose tissue (87, 221), suggesting that the adipose tissue-enriched IL-10-producing MAIT cells in humans could comprise a distinct subset (20).

### Cytotoxic Activity

In addition to cytokine secretion, MAIT cells and iNKT cells display direct cytotoxic activity. MAIT cell killing is mediated *via* the Perforin/Granzyme pathway and is independent of Fas/FasL and NKG2D (91, 161, 204). While their cytotoxic capacity (i.e., Perforin and Granzyme expression) is enhanced upon activation *via* the TCR and/or with cytokines, target cell killing is MR1 dependent (Figure 2) (91, 161, 204). In contrast to MAIT cells, iNKT cell killing can be mediated *via* both Perforin/Granzyme- and Fas/FasL-dependent pathways (Figure 2) (62, 98, 222). The cytotoxic capacity of iNKT cells varies between subsets. Human CD4^−^ iNKT cells show increased expression of cytotoxic molecules and superior cytotoxic activity compared with CD4^+^ cells (62, 223, 224). Whether CD4^+^, CD8^+^, and DN MAIT cells show differences in cytotoxic activity is currently unknown. Akin to MAIT cells, iNKT cell cytotoxicity is largely dependent on CD1d and antigen, although alternative CD1d-independent mechanisms have been described (202, 222, 224). In particular, human CD4^−^ iNKT cells can kill targets through a CD1d-independent NKG2D-dependent pathway (202). MR1-independent pathways for MAIT cell killing have yet to be identified.

### Immune Interactions

While knowledge of MAIT cell crosstalk with other immune cell subsets remains relatively limited, recent studies have identified important interactions with a number of immune cell types, including DCs, B cells, and NK cells. Through contact with myeloid cells, MAIT cells appear to have important immune regulatory functions. For example, upon antigen-specific activation, MAIT cells upregulate CD40L and induce CD40-dependent DC maturation, and in synergy with TLR ligands, promote the secretion of IL-12 (225). DC-derived IL-12 can subsequently enhance MAIT cell activation. MAIT cells can also influence monocyte differentiation *in vivo*. MR1^−/−^ mice show enhanced susceptibility to pulmonary infection with *Francisella tularensis* live vaccine strain and delayed bacterial clearance (44). In this setting, MAIT cells promote early GM-CSF production in the lungs, resulting in the differentiation of inflammatory monocytes into DCs and the recruitment of activated CD4^+^ T cells into the lungs (44, 226). In addition to their effects on myeloid populations, MAIT cells can provide non-cognate B cell help *in vitro* (227). In response to TCR-dependent or TCR- and cytokine-dependent activation, MAIT cells secrete factors that act on B cells to promote the differentiation of memory cells into plasmablasts and to increase antibody production (227). In these experiments, TCR stimulation was essential for the capacity of MAIT cells to provide B cell help (227). Finally, in the context of whole blood, activated MAIT cells promote NK cell transactivation in an MR1- and IL-18-dependent manner (225). Although MAIT cells can facilitate monocyte differentiation *in vivo*, whether the described crosstalk with DCs, B cells, and NK cells, occurs *in vivo*, and exactly where such interactions would take place, remains to be determined.

More is known about the immune interactions of iNKT cells. Through crosstalk with an array of immune cell types, iNKT cells can profoundly influence the nature and quality of both innate and adaptive immunity. iNKT cells engage in similar interactions to those described for MAIT cells; however, differences can be identified in the requirements for MAIT/iNKT cell activation in these settings and in the downstream effects on the immune response. Human iNKT cell clones drive monocyte differentiation in a CD1d-dependent manner (228). By contrast, MAIT cell-mediated monocyte differentiation is MR1-independent, at least for transgenic MAIT cells *in vitro* (226). Analogous to MAIT cells, bidirectional interaction between iNKT cells and DCs leads to DC maturation and NK cell transactivation, but also results in increased peptide-specific CD4^+^ and CD8^+^ T cell responses (229–232). A similar function has yet to be described for MAIT cells. In addition to non-cognate B cell help, iNKT cells can provide cognate B cell help (233). In some settings, help is provided by a dedicated subset of iNKT cells, known as follicular helper NKT (NKT_FH_) cells (93, 94, 234). Other specialized iNKT cell subsets also engage in key immune interactions. For example, mouse and human Foxp3^+^ invariant regulatory NKT (iNKT_reg_) cells have been shown to suppress naïve T cell proliferation *in vitro* (95, 96). Whether comparable functions can be performed by specialized MAIT cell subsets is currently unknown.

In summary, iNKT cells and MAIT cells rapidly produce cytokines, exhibit cytotoxic activity, and can influence the function of both innate and adaptive immune cell populations. MAIT cells typically produce pro-inflammatory cytokines, whereas iNKT cells secrete vast amounts of both pro- and anti-inflammatory cytokines. While immunoregulation *via* cytokine secretion is the dominant function of iNKT cells, the relative importance of cytokine secretion versus cytotoxic activity for MAIT cells, is currently unknown. iNKT cells profoundly influence the immune response through their crosstalk with other immune cell subsets, and limited studies reveal similar interactions for MAIT cells. Given their abundance in humans and their rapid effector function, MAIT cells are likely key orchestrators of innate and adaptive immunity in humans.

## Subsets

Although human MAIT cells are thought to be largely homogeneous in phenotype and function, recent findings suggest that they may be more diverse than currently appreciated. Lepore et al. identified subpopulations of human MAIT cells with distinct cytokine secretion profiles (50), although whether these represent separate lineages is currently unknown. Human MAIT cells also show heterogeneous expression of certain NK cell-associated receptors, including CD56 and CD84, the expression of which correlates with their functional response to cytokine stimulation (209). Finally, MAIT cells in certain tissues exhibit altered cytokine production. For example, MAIT cells are skewed toward a Th17-like phenotype in female genital tract and express lower levels of the transcription factors PLZF and Eomes compared with peripheral blood MAIT cells (26), suggesting that they may comprise a distinct MAIT cell subset.

Mucosal-associated invariant T cells in humans can be CD4^+^, CD8^+^, or DN (Table 1) (16). Coreceptor expression appears to have little practical significance, particularly for CD8^+^ and DN MAIT cells (43, 235). However, limited studies have characterized the phenotype and function of the minor CD4^+^ population. Moreover, surrogate methods for MAIT cell identification were recently shown to poorly define CD4^+^ MAIT cells (43, 235), and thus findings from previous studies that used such approaches, require validation. Initial studies using the MR1/5-OP-RU tetramer indicate some disparity between CD4^+^ and CD8^+^/DN subsets, including differential expression of certain transcription factors (PLZF, Eomes), chemokine receptors (CCR4, CXCR6), adhesion molecules (CD56), and NK cell receptors (NKG2A, NKG2D) (43, 235). Nevertheless, the role of the CD4 coreceptor and whether CD4^+^ MAIT cells comprise a distinct subset with specific functionality is currently unknown. Unlike MAIT cells, human CD4^+^, CD8^+^, and DN iNKT cells show clear phenotypic, functional, and transcriptional differences (15, 61, 62, 84, 202, 224, 236–240). However, given the considerable heterogeneity within each of these populations (15), it is unlikely that they represent genuine iNKT cell subsets.

**Figure 3:**
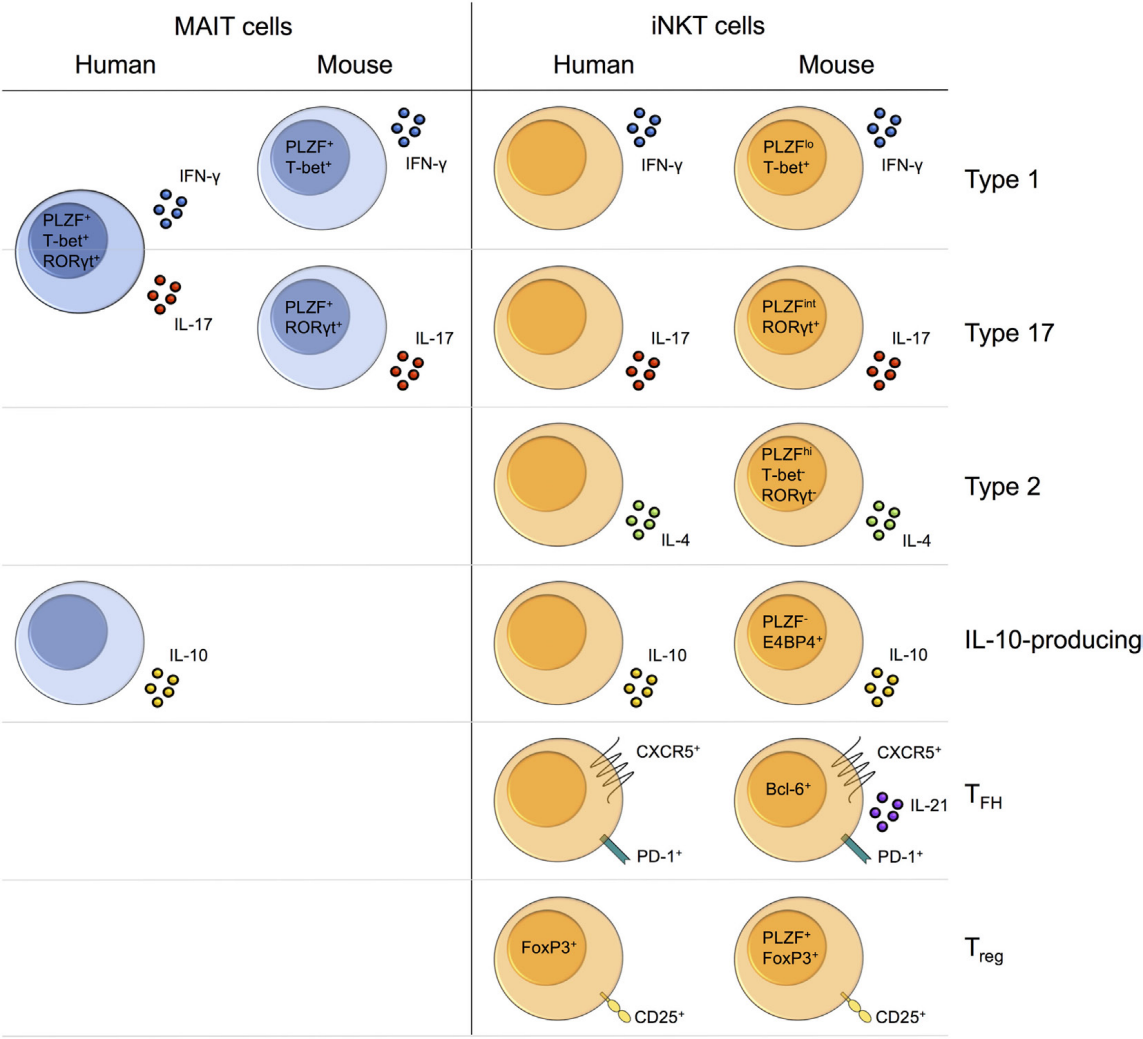
Functional capacity of mucosal-associated invariant T (MAIT) cells and invariant natural killer T (iNKT) cells. Where subsets of MAIT or iNKT cells have been defined, characteristic cytokines, transcription factors, and/or surface markers, are illustrated. MAIT cells and iNKT cells exhibit overlapping functions, although a wider range of functions have been described for iNKT cells. In mice, distinct type 1 and type 17 MAIT and iNKT cell subsets have been identified. By contrast, human MAIT cells exhibit a mixed type 1/type 17 phenotype. Human iNKT cells secrete IFN-γ and IL-17 (only *in vitro* under pro-inflammatory conditions), but whether these cytokines are produced by distinct subsets, remains to be established. Unlike MAIT cells, iNKT cells also show type 2 functions, such as IL-4 secretion. In mice, IL-10-producing iNKT cells comprise a distinct subset with altered transcription factor expression. Human MAIT cells and iNKT cells can produce IL-10, and human IL-10-producing MAIT cells are enriched in adipose tissue, similar to mouse NKT10 cells. However, whether these IL-10-producing populations comprise distinct subsets, is currently unknown. Finally, multiple specialized subsets of iNKT cells have been identified in mice, including NKT_FH_ cells and iNKT_reg_ cells. Human iNKT cells with similar phenotypes and/or functions have also been identified (NKT_reg_ only *in vitro*), but analogous populations have yet to be described for MAIT cells.

The evidence for MAIT cell subsets in mice is more convincing. Two subsets of MAIT cells, a major RORγt^+^IL-17^+^ population, and a smaller T-bet^+^IFN-γ^+^ subset have been identified in the thymus, spleen, and lung (Figure 3) (19, 102). These subsets can show plasticity *in vivo*. Following intranasal infection with *Salmonella. enterica* serovar Typhimurium, RORγt^+^ MAIT cells in the lung upregulate T-bet expression to become RORγt^+^T-bet^+^ cells that can secrete either IL-17 or IFN-γ, though the majority produce IL-17 (32). In contrast to mice, human thymic and peripheral blood MAIT cells coexpress RORγt and T-bet, although T-bet expression can similarly increase upon activation (85, 91, 102, 167). Furthermore, the majority of human MAIT cells express IFN-γ and a smaller fraction produce IL-17, while some secrete both cytokines (Figure 3), highlighting important differences between mouse and human MAIT cells (14).

Similar to MAIT cells, iNKT cells in mice differentiate into distinct subsets within the thymus. These mirror conventional CD4^+^ T helper cell subsets in their expression of master transcription factors and cytokines, namely PLZF^lo^T-bet^+^RORγt^−^ NKT1 cells that secrete IFN-γ, PLZF^hi^T-bet^−^RORγt^−^ NKT2 cells that secrete IL-4 and other Th2 cytokines, and PLZF^int^T-bet^−^RORγt^+^ NKT17 cells that secrete IL-17A and IL-22 (Figure 3) (86, 241). NKT1, NKT2, and NKT17 cells show highly divergent transcriptional programs and differ in their expression of chemokine receptors, NK cell receptors, cytotoxic molecules, and cell cycle-related genes (146, 147, 242). T-bet^+^IFN-γ^+^ and RORγt^+^IL-17^+^ MAIT cells in mice could represent “MAIT1” and “MAIT17” subsets (19, 102), akin to NKT1 and NKT17 cells, respectively. By contrast, no “MAIT2” subset comparable to NKT2 cells has been identified, and MAIT cells in mice and humans show little to no production of Th2 cytokines (14, 19).

In addition to the major iNKT cell subsets that develop in the thymus, a number of minor, highly specialized subsets have been identified in mouse peripheral tissues and/or lymphoid organs, but not in the thymus. These include NKT_FH_ cells (93, 234), iNKT_reg_ cells (95), and IL-10-secreting NKT10 cells (87, 221). However, it is important to note that IL-10 can also be produced by activated NKT1, NKT2, and NKT17 cells following stimulation with αGalCer (243). Human iNKT cells with a similar phenotype and/or function to NKT_FH_ cells (93), iNKT_reg_ cells (95, 96), and NKT10 cells (87) have been reported (Figure 3). By contrast, comparable MAIT cell subsets have not been defined in mice or humans, although a high proportion of MAIT cells secrete IL-10 in human adipose tissue (Figure 3) (20). As NKT10 cells are enriched in mouse adipose tissue (87, 221), IL-10-secreting MAIT cells could represent a dedicated “MAIT10” subset. Whether MAIT cells can have regulatory or follicular helper-type functions is currently unknown.

In brief, two distinct MAIT cell subsets, analogous to NKT1 and NKT17 cells, are present in mice. Whereas the MAIT cell population is biased toward RORγt/IL-17 expression in C57BL/6 mice, iNKT cells favor the expression of T-bet/IFN-γ. This could suggest functional segregation between innate-like T cell populations in mice, although depending on the tissue and mouse strain, NKT17 cells might still be more abundant than MAIT cells. In comparison with mouse MAIT cells, human MAIT cells appear more homogeneous and exhibit a mixed Th1/Th17 phenotype, although there is evidence for some phenotypic and functional diversity. Moreover, human MAIT cells preferentially secrete IFN-γ as opposed to IL-17. Multiple iNKT cell subsets are present in both mice and humans, although they are better defined in mice. Compared with MAIT cells, iNKT cells appear more functionally diverse, although it is possible that additional MAIT cell subsets remain to be identified.

## Avenues for Future Mait Cell Research

With the recent generation of MR1 tetramers (12, 13), it is now possible to detect MAIT cells in wild-type mice (19), as well as in human settings where MAIT cell frequency is low, for example in the thymus (102). Consequently, MAIT cells are now being studied in an increasingly wide variety of settings, including in numerous human diseases and animal disease models. Undoubtedly, this will lead to a greatly improved understanding of the role of MAIT cells in both health and disease.

Compared with iNKT cells, our knowledge of MAIT cell phenotype, development, regulation, and function remains limited and there are many important questions that need to be addressed. The search for novel MAIT cell ligands, both for their selection in the thymus, and their activation in the periphery, is a particularly active area of investigation. It is currently unknown whether MAIT cells can recognize endogenous antigens, analogous to iNKT cells, and if so, how these might influence MAIT cell development and function *in vivo*.

There are many gaps in our understanding of MAIT cell development, including the signals required for positive and potentially negative selection in the thymus; the transcriptional and epigenetic regulation of MAIT cell differentiation; and the timing and location of MAIT cell maturation. As MAIT cells appear to be mature in fetal tissues (155), but naïve in cord and neonatal blood (16, 47), they may comprise predominantly tissue-resident populations, similar to iNKT cells (28, 29, 221). This could be addressed in parabiosis studies. Moreover, it will be important to determine the signals governing peripheral MAIT cell maturation and the maintenance of homeostasis. In addition to IL-18 and IL-23 (77, 102), it is worth investigating the role of IL-7 and IL-15, given MAIT cell responsiveness to these cytokines (30, 91, 167–169) and their function in iNKT cell homeostasis (73, 75, 76, 166, 244). Whether MR1 is required for peripheral MAIT cell survival and maintenance *in vivo* is currently unknown, although CD1d does not appear to be essential for the homeostasis of iNKT cells (75, 142, 166, 170).

While MAIT cells can be activated through the TCR or with cytokines, or by a combination of both (11), which of these activation mechanisms predominates *in vivo* remains to be determined. iNKT cell activation appears to be dominantly driven by cytokines, even in settings where microbial ligands are present (78), although their sensitivity to cytokines alone may require prior TCR priming (79). It is hypothesized that cytokines might also be key for MAIT cell activation, especially given the indiscriminate expression of their ligands in both commensal and pathogenic bacteria (7, 17), and their relative hyporesponsiveness to TCR stimulation (30, 85, 169). Despite expression of various NK cell receptors, costimulatory molecules, and immune checkpoint molecules (14, 209), how stimulation of these receptors modulates MAIT cell activation, is essentially unknown.

Similar to iNKT cells (86), transcriptionally and functionally distinct MAIT cell subsets have been identified in mice (19), although these require further characterization. Human MAIT cells exhibit a relatively uniform phenotype, and coexpress key transcription factors, suggesting a single MAIT cell subset (14, 91). However, recent studies indicate previously unappreciated phenotypic and functional heterogeneity in blood MAIT cells (14, 50, 209), as well as skewed or unique functions in certain tissues (20, 26, 155). Thus, the existence of specialized MAIT cell subsets remains an open question. In addition, it is unclear to what extent MAIT cells can show functional plasticity driven by environmental factors. It is likely that the presence of subsets and the ability of MAIT cells to display functional plasticity both contribute to MAIT cell diversity.

Recently, a number of studies have increased our knowledge of MAIT cell interactions with other immune cell populations (225–227), although our understanding of MAIT cell crosstalk remains much more limited than for iNKT cells (9). Furthermore, the role of MAIT cell interactions *in vivo* and their influence on the overall innate and adaptive immune response is largely unknown. As the interactions identified for MAIT cells resemble those of iNKT cells, it may be possible to use known iNKT cell interactions as a framework for the further investigation of MAIT cell crosstalk.

Though outside the scope of this review, we have little understanding of the role of MAIT cells in disease, despite their altered frequency and function in numerous infectious, inflammatory, and malignant diseases (1, 4, 245). Given that MR1 tetramers are now widely available, it is possible to address the role of MAIT cells *in vivo* in models of disease. Recently, MR1 ligands have been discovered among drugs and drug-like molecules (59). This indicates the potential for the future development of therapeutics to modulate MAIT cell function (59), akin to the development of αGalCer as a stand-alone therapy or vaccine adjuvant (246).

## Conclusion

Invariant natural killer T cells have been more extensively studied than MAIT cells due to their higher frequency in mice, the earlier development of tetramers for their specific identification, and the earlier generation of relevant transgenic mouse models. However, the MAIT cell field is growing rapidly, due to the recent development of the MR1 tetramer and hence the possibility to examine MAIT cells in wild-type mice. Comparison of MAIT cells to iNKT cells has in many cases identified shared biology. However, there are also instances in which comparison of MAIT cells to iNKT cells has revealed distinct biology. Therefore, while iNKT cell research provides a useful framework for the study of MAIT cells, it is important that both populations are studied individually, and findings from the iNKT cell field are not presumed to also apply to MAIT cells. Given their abundance in humans, their capacity for rapid cytokine production in response to TCR and/or cytokine stimulation, and their interactions with other immune cell populations, MAIT cells are likely key players in the immune system, both in health and disease. In support of this, they show altered phenotype and function in numerous human diseases, and exhibit protective or deleterious roles in mouse models of infection or inflammation. Consequently, MAIT cells represent an attractive target for therapeutic manipulation, especially considering their high frequency in humans and their recognition of a monomorphic MHC-like molecule. To realize this goal, further research is necessary to develop a greater understanding of MAIT cell development, function, and regulation, and their specific roles in disease. We should continue to leverage our accumulated knowledge of iNKT cell biology as a platform to more completely understand the unique, and shared, biology of MAIT cells. In doing so, such investigations will likewise enhance our understanding of iNKT cell biology.

## Author Contributions

LG wrote the bulk of the manuscript and contributed to the figures. PK contributed to the planning, editing, and scope of the review. NP edited and revised the manuscript, and created the figures.

## Conflict of Interest Statement

The authors declare that the research was conducted in the absence of any commercial or financial relationships that could be construed as a potential conflict of interest.
